# PINS’s Commitment to Research and Academic Excellence

**DOI:** 10.12669/pjms.40.12(PINS).11413

**Published:** 2024-12

**Authors:** Asif Bashir

**Affiliations:** Asif Bashir, MD, FAANS, FACS Diplomate American Board of Neurological Surgery Professor & Chair Neurosurgery Executive Director & Dean Punjab Institute of Neurosciences (PINS) Lahore General Hospital (LGH) Lahore, Pakistan. Email: asifbashirMD@gmail.com

It is with immense pride and gratitude that we write this editorial for the *PINS-PJMS Supplement 2024*. This publication marks a significant milestone for the Punjab Institute of Neurosciences (PINS), and I am thrilled to see our commitment to research and academic excellence come to fruition. Despite being an institution that is often understaffed and underfunded, we have immense potential—potential that is brought to life in this supplement through the hard work, dedication, and expertise of everyone involved. This supplement is a testament to the talent and resilience we have at PINS, and I’m deeply proud of what we have accomplished.

Research and innovation are critical in the field of medicine, especially in neuroscience. This supplement highlights the important strides we are making to bridge the gap between where we are academically and where we aspire to be. With the invaluable support of the *Pakistan Journal of Medical Sciences*, we are moving closer to our goal of making PINS a leader in both research and quality patient care in neurosciences across the region. It is through collaborations like these that we continue to grow, ensuring our work reaches the highest standards of excellence.

Despite the challenges that we face, our deeply inculcated culture of unity and perseverance is what will carry us into the future. Our patient base is vast, and we are regularly faced with complex neurological and spine pathology that demand advanced solutions. Our physicians, researchers, and healthcare professionals possess the skills and dedication necessary to elevate this institution to international standards. PINS aims to provide the same level of care found in the US, Europe, and other advanced healthcare systems to every patient, regardless of their financial background. We take pride in treating both the affording and the poor with equal care and compassion. We also recognize that public-private support is essential to achieving our goals. PINS must serve as a hub for all neuro-spine subspecialties under one roof, offering world-class care and research while ensuring equitable healthcare for all.

We are well on our way to realizing this vision. From the establishment of our editorial office, which will be inaugurated later this year to support our local publication, Pakistan Journal of Neurological Surgeons (PJNS), to the introduction of innovative programs such as visiting professor collaborations featuring the brightest minds in the world, PINS is making significant strides toward academic and clinical excellence. Our largest postgraduate residency training programs, rooted in the proven pedagogical methods, continue to train the next generation of neurosurgeons, neurologists, neuro anesthesiologists & neuro radiologists.

This achievement would not have been possible without the unwavering support of *Pakistan Journal of Medical Sciences* and the dedication of our national and international reviewers whose time, expertise and critical feedback have played a crucial role in the success of this publication. Our neurosurgery resident Dr. Haseeb Mehmood Qadri needs a special mention who, through persistent commitment and untiring efforts, transformed an idea into a remarkable reality.

We would like to extend our heartfelt thanks to PJMS for providing us with a platform to share our research and ideas. A special thanks goes to Mr. Shaukat Ali Jawaid, Chief Editor PJMS, a dear friend and colleague, whose guidance and support have been instrumental in making this supplement come into existence. Together, we will continue to push forward, ensuring PINS stands as a beacon of excellence, compassion, and innovation—a place where every patient is treated with the utmost care and every discovery paves the way for a brighter future in neurological and spine care.

Sincerely,

